# Pediatrician-experienced barriers in the medical care for refugee children in the Netherlands

**DOI:** 10.1007/s00431-018-3141-y

**Published:** 2018-04-20

**Authors:** A. Baauw, S. Rosiek, B. Slattery, M. Chinapaw, M. Boele van Hensbroek, J. B. van Goudoever, J. Kist-van Holthe

**Affiliations:** 1 Department of Pediatrics, Rijnstate Hospital, Wagnerlaan 55, 6816 AD Arnhem, The Netherlands; 2 Department of Pediatrics, VU University Medical Center and Emma Children’s Hospital - Academic Medical Center, University of Amsterdam, Amsterdam, the Netherlands; 3 Amsterdam Public Health Research Institute, Department of Public and Occupational Health, VU University Medical Center, Amsterdam, the Netherlands

**Keywords:** Asylum seekers, Relocations, Screening of refugee children, Health status, Medical records, Health literacy, Unaccompanied refugee minor

## Abstract

Pediatricians in the Netherlands have been confronted with high numbers of refugee children in their daily practice. Refugee children have been recognized as an at-risk population because they may have an increased burden of physical and mental health conditions, and their caretakers may experience barriers in gaining access to the Dutch health care system. The aim of the study was to gain insight into the barriers in the health care for refugee children perceived by pediatricians by analyzing logistical problems reported through the Dutch Pediatric Surveillance Unit, an online system where pediatricians can report predefined conditions. Pediatricians reported 68 cases of barriers in health care ranging from mild to severe impact on the health outcome of refugee children, reported from November 2015 till January 2017. Frequent relocation of children between asylum seeker centers was mentioned in 28 of the reports on lack of continuity of care. Unknown medical history (21/68) and poor handoffs of medical records resulting in poor communication between health professionals (17/68) contributed to barriers to provide good medical care for refugee children, as did poor health literacy (17/68) and cultural differences (5/68).

*Conclusion*: Frequent relocations and the unknown medical history were reported most frequently as barriers impacting the delivery of health care to refugee children. To overcome these barriers, the Committee of International Child Health of the Dutch Society of Pediatrics recommends stopping the frequent relocations, improving medical assessment upon entry in the Netherlands, improving handoff of medical records, and improving the health literacy of refugee children and their families.

**Table:** 

**What is Known:** • *Pediatricians in the Netherlands are confronted with high numbers of refugee children* • *Refugee children represent a population that is especially at risk due to their increased burden of physical and mental health conditions*
**What is New:** • *Refugee children experience barriers in accessing medical care* • *To start overcoming these barriers, we recommend that frequent relocations be stopped, health assessment upon entry in the Netherlands be improved, medical handoffs be improved, and that the refugees be empowered by increasing their health literacy*

## Introduction

### Refugees in a global perspective

The global population of forcibly displaced persons has substantially increased over the last two decades from 37.3 million individuals in 1996 to 65.6 million in 2016 [15]. Throughout 2016, 1.2 million new refugees and asylum seekers arrived in Europe, bringing the total number of refugees living on the continent to just under 4.4 million [7]. Complicating this global humanitarian situation is the fact that about half of all refugees and asylum seekers are children. According to Eurostat, nearly 400,000 children claimed asylum in Europe in 2016 and 15.9% of these children registered as unaccompanied minors [7].

Box 1 Definitions

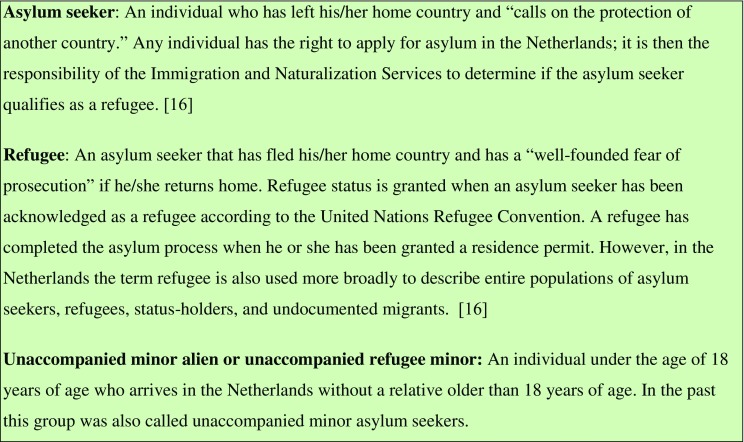



### Refugees in the Netherlands

With a population of 17 million people, the Netherlands holds the sixth largest number of refugees in Europe. In 2016, there were 101,702 officially recognized refugees and an additional 12,245 asylum seekers with pending applications [8] (Tables 1). The number of unaccompanied minors arriving in the Netherlands has also rapidly increased in recent years. In 2015, 3859 unaccompanied minors registered throughout the country, and in 2016, an additional 1536 unaccompanied minors registered, a marked increase from the 962 applications submitted in 2014 [14]. The Central Agency for the Reception of Asylum Seekers provides accommodation for asylum seekers during the asylum procedure. Asylum seekers in the Netherlands are randomly allocated to the 61 centers with a capacity for 31,000 asylum seekers (end 2017). Asylum seekers are moved between various asylum seeker centers as part of the asylum process. They can also be relocated as a result of closure of a location, as a sanction, or on request. The annual relocation rates vary from one to four relocations per year, and on average is one relocation per asylum seeker per year. Asylum-seeking children have special rights and are not kept in custody. They are entitled to an education [9].

**Table 1:** Total asylum requests, unaccompanied minor refugees, and total live births in the Netherlands [14]

	Total asylum requests	Children and adolescents	UMR*	Total live births Netherlands
2013	16,725	5860	301	171,341
2014	29,890	8700	962	175,181
2015	58,880	19,010	3859	170,510
2016	31,600	12,705	1536	172,520

*Unaccompanied minor refugee

### Public and curative health care services for refugee children in the Netherlands

Upon arrival in the Netherlands, refugee children receive a non-mandatory health assessment by a Child Health Care doctor and nurse. These medical examinations are the responsibility of the Central Organization of Asylum seekers when it concerns asylum seekers and of the municipalities when it concerns children with a residence permit and children entering the Netherlands in the context of family reunification. The aim of this intake is to evaluate the health status and psychological well-being of a child as well as to make an inventory of vaccination status and, if needed, provision of additional vaccination(s). The medical health screening includes taking a medical history and a physical examination, but does not include any laboratory tests. The Tuberculosis Department of the Public Health Department checks the children from high-prevalence countries as defined by the World Health Organization for active tuberculosis with a chest X-ray as soon as the child is able to sit upright [18]. When a child is ill, the curative medical services in the asylum seeker centers provide the first-line care. If needed, children are referred to a pediatrician, psychiatrist, or other specialist [2].

Refugee children are at an increased risk for a variety of conditions, including anemia, hemoglobinopathies, hepatitis B, (latent) tuberculosis, intestinal parasites, malaria, the human immunodeficiency virus (HIV) infection, malnutrition, and micronutrient deficiencies [12, 20]. The vulnerable status of refugee children is compounded by the unfavorable conditions in their home countries, the migration itself, and the stress of living in unfamiliar surroundings [3, 10, 19]. This vulnerability can manifest in an increased burden of physical and mental health conditions and disparities in order to gain urgent access to care (health-seeking behavior) [5].

#### Aim of the study

The aim of the study was to gain insight into the barriers in the health care for refugee children perceived by pediatricians by analyzing logistical problems reported by pediatricians through the Dutch Pediatric Surveillance Unit.

## Methods

To gain insight into the mechanisms responsible for impediments in health care for refugee children, a report system was used—the Dutch Pediatric Surveillance Unit. The Dutch Pediatric Surveillance Unit was founded in 1992 and is part of the Dutch Pediatric Association. All pediatricians in the Netherlands, working in a clinical setting (*n* = approximately 1300), were asked on a monthly basis to register a small number of diseases in children through a web-based system. On 1 November 2015, logistical problems in the care for refugee children were added to the recording system. Data collected in the first year of recording were analyzed and reported in this manuscript. Registration was performed anonymously. Descriptive qualitative thematic analysis by two independent researchers was used to analyze the reports.

## Results

Pediatricians reported 74 cases of logistical problems with refugee children. After removal of six duplicates, 68 cases were analyzed (the same child was reported by more than one pediatrician). Often, more than one barrier per report was described. Descriptive thematic analysis was used to analyze the DPSU reports. Each report was read carefully to deeply understand what was mentioned and to identify the main problem. Afterwards, each report was labeled in order to create themes. The problems mentioned in the reports were divided into five themes: frequent relocations, lack of previous medical records/information, poor handoffs of medical records, poor health literacy, and cultural differences (Tables 2).

**Table 2:** Barriers in the health care for refugee children detected by pediatricians in the Netherlands

Barriers	*N*
Frequent relocations	28
Unknown medical history	21
Poor handoffs of medical records	17
Poor health literacy	17
Cultural differences	5

### Frequent relocations

In 28 of the cases, relocation of refugee children to another asylum seekers center was associated with problems of health care delivery. The impact of the disruption in the continuity of care ranged from failing to attend a follow-up appointment, to missing a planned—and sometimes life-saving—treatment.

Box 2 Examples of cases of disruption of continuity of care due to frequent relocations

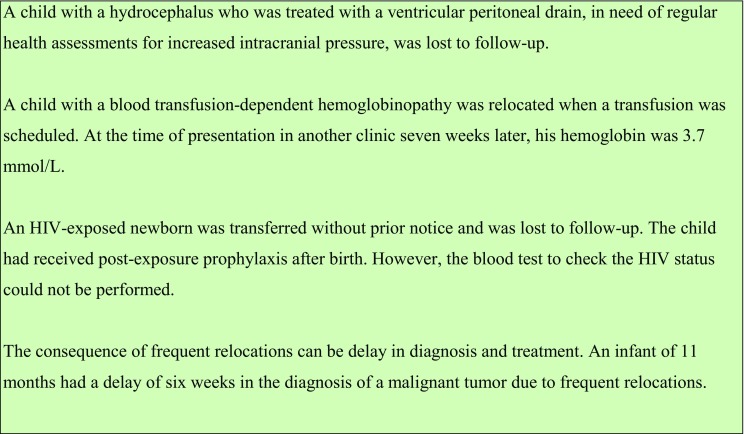



### Unknown medical history

Concerns about unknown medical history were reported in 21 cases.

Refugee children usually enter the Netherlands without any medical record from their country of origin. Records may have been lost during the flight or simply are unavailable. The health screening performed by Child Health Care relies solely on oral information provided by parents, caretakers, or the children themselves. This is especially a problem in unaccompanied refugee minors, who often do not know their medical history.

Box 3 Examples of cases of unknown medical history in refugee children

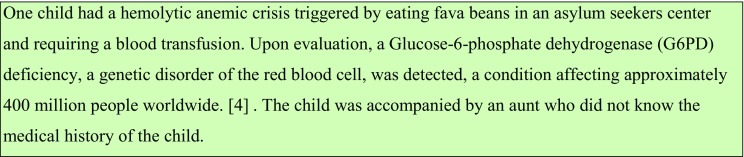



### Poor handoffs of medical records

Poor outcomes or complications due to the lack of a central medical record form were reported 17 times. Many different Health Information Systems are used by the public and curative health services in the Netherlands and often these systems are not connected. The electronic patient document is not stored in an accessible database, with the exception of the tuberculosis control program. Because the medical record of the initial health assessment by the Youth Health Care service is not available when a child presents in the hospital, frequent lack and loss of essential information occurs, especially in cases where caregivers are not available to provide information.

Box 4 Examples of poor handoffs of medical records

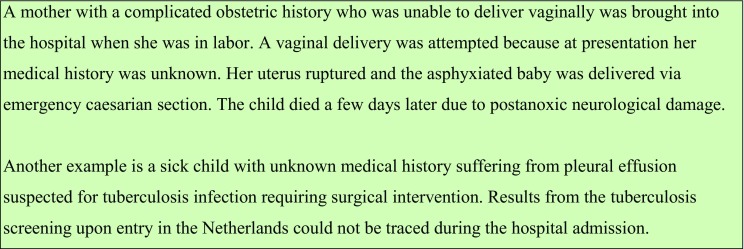



### Poor health literacy

The ability to read information and instructions as well as to understand the health systems is defined as a person’s health literacy [17]. Determinants for effective patient-doctor communication are at individual, collective, and health system level. The impact of poor health literacy on the access to, and quality of, care was reported 17 times. Errors with medication were reported frequently. Prescriptions are not understood, and allergies are not communicated, resulting in medication errors leading to toxic doses and severe allergic reactions.

Box 5 Examples of cases of poor health literacy

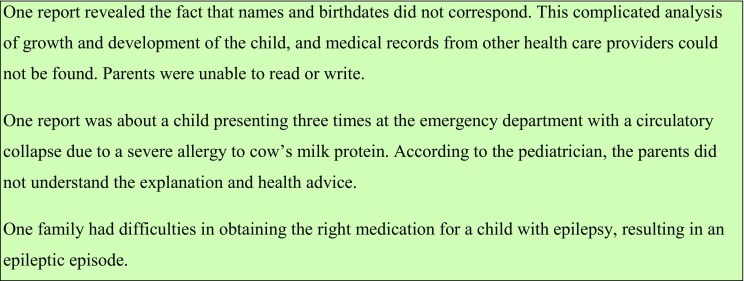



### Cultural differences

Cultural background is one of the determinants of (the perception) of health and disease [11]. Five cases described how cultural background defined the presenting symptoms and perceived health needs. Children with a mental problem including post-traumatic stress disorders often present with physical complaints and are often not recognized as such. Because the presenting symptoms are often culture-dependent, the perceived health needs of the patient and the health care worker do not always correspond.

Box 6 Examples of a case due to differences in cultural background

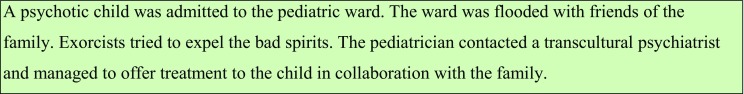



## Discussion

Despite the fact that health care services in the Netherlands are of a high standard, pediatricians experienced multiple barriers in delivering health care to refugee children. Levesque defines access to health care as the opportunity to reach and obtain appropriate health care services in situations of perceived need for care [11]. Access to health care is viewed as the possibility to identify health care needs, to seek health care services, to reach the health care resources, to obtain or use health care services, and to actually be offered services appropriate to the needs for care. Access is at the interface between the characteristics of persons, households, social and physical environments, and the characteristics of health systems, organizations, and providers. The supply side and demand side are interdependent [11].

Box 7 Levesque 2013: a conceptual framework of access to health care [11]

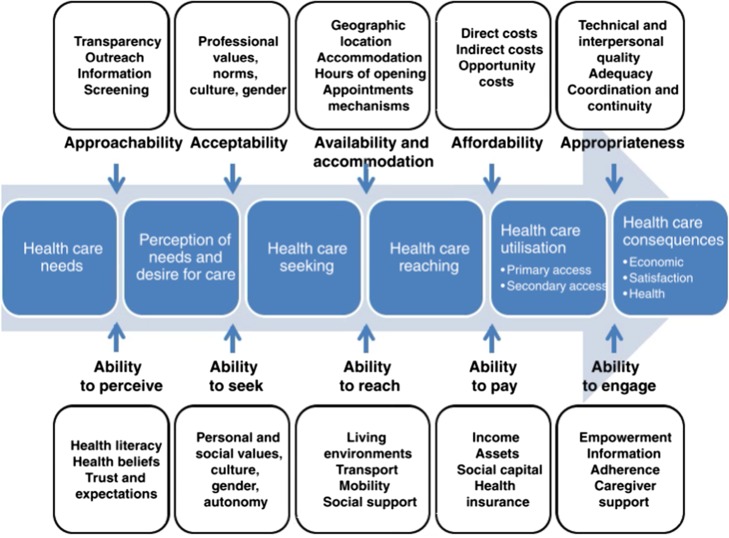



Frequent relocations of refugee children in the asylum procedure were associated with disruption of the continuity of medical care for refugee children. Children with a complex medical problem requiring intensive or a multidisciplinary approach were most vulnerable for this disruption. Frequent relocations are the result of policies and regulations. But not all relocations result in the loss of continuity of care. Individual factors like high-health literacy can help to overcome the barriers.

Refugee children presenting in the consultation room of the pediatrician often have an unknown medical history. Unknown medical history is determined by internal and external factors, at both the individual and community level. In several cases, the absence or loss of medical records resulted in potentially life-threatening complications. Norms and values of the health care professionals, the communication skills of the individual, the culture within a health care organization, and the organization of care all contribute to the quality of communication between health professionals and between patient and doctor [1]. In the intercultural context, medical communication is a challenge, with the health systems or the health information provided not always being fully understood [17]. Health literacy and culture influence the patient-doctor relationship and the linkage to the health systems [13]. Individual internal factors such as the health beliefs of the individual, and external factors, such as the demographics combined with external factors at community level, i.e., health insurances, influence how refugees perceive their health needs. The use of a professional translator or interpreter can help to overcome inadequate language concordance, but does not necessarily lead to adequate patient-doctor communication. In a substantial number of cases, the barriers described resulted in a delay of care.

Personal involvement and empathy of pediatricians with interest in this specific group might have contributed to creative solutions that helped overcome system constraints. For example, seven of 21 pediatricians reported that a relocated child could be traced to another asylum seeker center so that proper treatment could be rescheduled. However, this requires dedication as it is a time-consuming exercise that involves directly contacting the Central Agency for the Reception of Asylum Seekers.

A positive impact of the Dutch Pediatric Surveillance Unit reports is the awareness it created among policymakers at the Ministry of Health and the Central Agency of Asylum Seekers. The collection of these reports directly contributed to a policy change that exempted children with complex medical needs from being relocated in specific cases. At the time of this paper’s submission, four children have been excluded from relocation and were intentionally placed near a university hospital that could adequately meet their needs—three patients with Sickle cell anemia disease and one patient with a connective tissue disorder which requires specialized cardiac services. In addition, these reports also lead to a nationwide policy change which prohibited fava beans from being served in asylum seeker centers after a pediatrician reported the adverse reaction of an asylum-seeking child with G6PD (Box 3) [6].

### Strengths and limitations

The Dutch Pediatric Surveillance Unit is strongly embedded in the daily practice of all pediatricians in the Netherlands and has been a valuable tool in the field of Dutch pediatrics for more than 25 years. This strong history contributed to the strength of this study’s findings. However, the main limitation of the study is the voluntary participation in the surveillance system, and conclusions should be viewed in this perspective. Furthermore, this study is limited by its qualitative nature. Quantitative data such as the national incidence and prevalence rates of specific problems reported could not be obtained.

## Conclusion

Refugee children are a particularly vulnerable group and have increased health risks. In the Netherlands, pediatricians experience several barriers to the health care delivery that hinder the accessibility and quality of health care for refugee children. The main impediments are frequent relocations, unknown or incomplete medical history, poor handoffs of medical records leading to impaired communication between health care workers, low-health literacy of refugee children and their caretakers, and cultural differences.

### Recommendations

The Section International Child Health of the Dutch Society of Pediatrics strongly advises against the frequent relocations of refugee children in the asylum procedure and advocates improving the health assessment of refugee children upon entry in the Netherlands.

Box 8 Recommendations

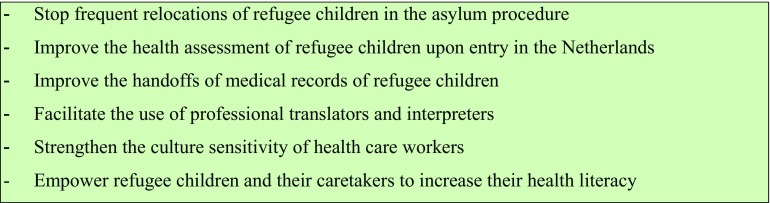



Our expectation is that many of the refugee children will be granted permanent residency and become an integral part of Dutch society. These children have the same health rights and the same right to access care as Dutch citizens. This study serves as a starting point for further collaboration between health care providers and policy makers. Together we must begin addressing the barriers to the medical care of refugee children experienced by pediatricians and work to ensure that all refugee children have access to health care at the appropriate level.

## Abbreviations

HIV: Human immunodeficiency virus
G6PD: Glucose-6-phosphate dehydrogenase
